# A Treatise on Insanity, and Other Disorders Affecting the Mind

**Published:** 1835-04-01

**Authors:** 


### MEDICAL QUARTERLY REVIEW.

### REVIEWS.

*A Treatise on Insanity, and other Disorders affecting the Mind.* By JAMES COWLES PRICHARD, M.D., F.R.S., &c.—London, 1835. 8vo. pp. 483.

THE high reputation of Dr. Prichard awakens expectations which are not disappointed by the work before us. We proceed, without preface, to give the reader some idea of its contents.

The first chapter is introductory, and treats of the definition and nosography of insanity.

If any argument were required to prove that medicine cannot at present be numbered among the exact sciences, that argument might be found in the fact, that definitions—the very basis of all the exact sciences—are, generally speaking, impracticable in everything that relates to it; nay, further, that the attempt to frame them, so far from affording a firm basis of reasoning, has usually tended to retard the progress of knowledge, by excluding truths as unquestionable as those insisted upon; so that a medical definition is often at best but an insulation of a certain number of truths, to say nothing of its being occasionally an annunciation of opinions whose validity is more than doubtful. The definition of insanity has frequently exercised the ingenuity of medical writers, but to very little purpose; since we know of none hitherto proposed which may not be nullified by a moment's reflection. Dr. Prichard judiciously substitutes a general classification of the phenomena of insanity for a precise definition of the term, which is in effect a generic one, comprising species whose characters are mutable, and which is hence insusceptible of exact definition. Our author includes the principal forms or varieties of insanity under the following heads:

"1. *Moral Insanity*, or madness consisting in a morbid perversion of the natural feelings, affections, inclinations, temper,

( = no. 1, vol 4 )

habits, moral dispositions, and natural impulses, without any remarkable disorder or defect of the intellect, or knowing and reasoning faculties, and particularly without any insane illusion or hallucination.

"The three following modifications of the disease may be termed *Intellectual Insanity*, in contradistinction to the preceding form. They are severally:—

"2. *Monomania*, or partial insanity, in which the understanding is partially disordered or under the influence of some particular illusion, referring to one subject, and involving one train of ideas, while the intellectual powers appear, when exercised on other subjects, to be in a great measure unimpaired.

"3. *Mania*, or raving madness, in which the understanding is generally deranged; the reasoning faculty, if not lost, is confused and disturbed in its exercise; the mind is in a state of morbid excitement, and the individual talks absurdly on every subject to which his thoughts are momentarily directed.

"4. *Incoherence*, or dementia. By some persons it may be thought scarcely correct to term this a form of insanity, as it has been generally considered as a result and sequel of that disease. In some instances, however, mental derangement has nearly this character from the commencement, or at least assumes it at a very early period. I am therefore justified in stating it, after Pinel, to be a fourth and distinct form of madness. It is thus characterised by that justly celebrated writer: 'Rapid succession or uninterrupted alternation of insulated ideas, and evanescent and unconnected emotions; continually repeated acts of extravagance; complete forgetfulness of every previous state; diminished sensibility to external impressions; abolition of the faculty of judgment; perpetual activity.'" (P. 6.)

"If I am correct," continues Dr. Prichard, "in assuming that all the varieties of madness may find their place under one of the descriptions thus marked out, a short nosography of the disease, which will answer many of the purposes of a definition, will be furnished by summing up the characteristics of the different forms. We may, then, describe insanity as a chronic disease, manifested by deviations from the healthy and natural state of the mind, such deviations consisting either in a *moral perversion*, or a disorder of the feelings, affections, and habits of the individual, or in *intellectual derangement*, which last is sometimes partial, namely, in *monomania*, affecting the understanding only in particular trains of thought; or general, and accompanied with excitement, namely, in *mania*, or *raving madness;* or, lastly, confounding or destroying the connexions or associations of ideas, and producing a state of *incoherence*." (P. 7.)

This arrangement of mental disorders had been adopted by Dr. Prichard, before he had seen the work of Professor Heinroth, whose classification is similar in principle, but more

3

minute. Our author gives an outline of Heinroth's system, which he regards as the most complete that could be formed, but has found no reason for abandoning his own.

We do not quite agree with our author as to the perfection of the German Professor's arrangement, and think that Dr. Prichard has done quite right in retaining his own in preference. We regard Heinroth's definition of his second form as exceedingly faulty: "*Depression;* simple melancholy, dejection without illusion of the understanding." We cannot conceive any form of mental disease as properly included under this definition. If simple low spirits be meant, without the presence of any assignable cause of mental depression, half our ladies of quality are mad every forenoon; again, if it be meant that there is a state of mind in which the smallest evils cast an appalling shadow, we apprehend that such state always involves more or less disorder of the judgment, by which the relative importance of objects is incorrectly appreciated.

A man, for instance, feels his sensibility deeply and permanently wounded by every little unkindness on the part of his friends,—he is upbraided by his conscience for every small deviation from right, as if he had committed an enormous crime,—a slight pecuniary loss, which may really be of very little consequence, to him amounts to nothing less than utter ruin and beggary: in all these cases the judgment is perverted, not with reference to the fact, but to the importance to be attached to it. We never yet saw a case of melancholy in which there was not either an illusion created by the imagination, or a perversion of the judgment as to matters of fact.

The second chapter treats of the phenomena of insanity, as manifested in the different forms of the disease.

"*Moral Insanity.* This form of mental derangement has been described as consisting in a morbid perversion of the feelings, affections, and active powers, without any illusion or erroneous conviction impressed upon the understanding; it sometimes coexists with an apparently unimpaired state of the intellectual faculties.

"There are many individuals living at large, and not entirely separated from society, who are affected in a certain degree with this modification of insanity. They are reputed persons of a singular, wayward, and eccentric character. An attentive observer will often recognize something remarkable in their manners and habits, which may lead him to entertain doubts as to their entire sanity; and circumstances are sometimes discovered, on inquiry, which add strength to his suspicion. In many instances it has been found that an hereditary tendency to madness has existed in the family, or that several relatives of the person affected have laboured under other diseases of the brain. The individual himself has been discovered to have suffered, in a former period of life, an attack of madness of a decided character. His temper and dispositions are found to have undergone a change; to be not what they were previously to a certain time: he has become an altered man, and the difference has, perhaps, been noted from the period when he sustained some reverse of fortune, which deeply affected him, or the loss of some beloved relative. In other instances, an alteration in the character of the individual has ensued immediately on some severe shock which his bodily constitution has undergone. This has been either a disorder affecting the head, a slight attack of paralysis, a fit of epilepsy, or some febrile or inflammatory disorder, which has produced a perceptible change in the habitual state of the constitution. In some cases the alteration in temper and habits has been gradual and imperceptible, and it seems only to have consisted in an exaltation and increase of peculiarities, which were always more or less natural and habitual.

"In a state like that above described, many persons have continued for years to be the sources of apprehension and solicitude to their friends and relatives. The latter, in many instances, cannot bring themselves to admit the real nature of the case. The individual follows the bent of his inclinations; he is continually engaging in new pursuits, and soon relinquishing them without any other inducement than mere caprice and fickleness. At length the total perversion of his affections, the dislike, and perhaps even enmity, manifested towards his dearest friends, excite greater alarm. When it happens that the head of a family labours under this ambiguous modification of insanity, it is sometimes thought necessary, from prudential motives, and to prevent absolute ruin from thoughtless and absurd extravagance, or from the results of wild projects and speculations, in the pursuit of which the individual has always a plausible reason to offer for his conduct, to make some attempt with a view to take the management of his affairs out of his hands. The laws have made inadequate provision for such contingencies, and the endeavour is often unsuccessful. If the matter is brought before a jury, and the individual gives pertinent replies to the questions that are put to him, and displays no particular mental illusion, a feature which is commonly looked upon as essential to madness, it is most probable that the suit will be rejected.

"Persons labouring under this disorder are capable of reasoning or supporting an argument upon any subject within their sphere of knowledge that may be presented to them; and they often display great ingenuity in giving reasons for the eccentricities of their conduct, and in accounting for and justifying the state of moral feeling under which they appear to exist. In one sense, indeed, their intellectual faculties may be termed unsound; they think and act under the influence of strongly-excited feelings, and persons accounted sane are, under such circumstances, proverbially liable to error both in judgment and conduct." (P. 12.)

Moral insanity has been recognised by Pinel as a distinct form of derangement; he calls it "emportement maniaque sans délire." The following is a characteristic instance given by this author.

"'An only son of a weak and indulgent mother gave himself up habitually to the gratification of every caprice and passion of which an untutored and violent temper was susceptible. The impetuosity of his disposition increased with his years. The money with which he was lavishly supplied removed every obstacle to the indulgence of his wild desires. Every instance of opposition or resistance roused him to acts of fury. He assaulted his adversary with the audacity of a savage; sought to reign by force, and was perpetually embroiled in disputes and quarrels. If a dog, a horse, or any other animal offended him, he instantly put it to death. If ever he went to a fête or any other public meeting, he was sure to excite such tumults and quarrels as terminated in actual pugilistic rencon-ters, and he generally left the scene with a bloody nose. This way-ward youth, however, when unmoved by passion, possessed a perfectly sound judgment. When he became of age, he succeeded to the possession of an extensive domain. He proved himself fully competent to the management of his estate, as well as the discharge of his relative duties, and he ever distinguished himself by acts of beneficence and compassion. Wounds, law-suits, and pecuniary compensations, were generally the consequences of his unhappy propensity to quarrel. But an act of notoriety put an end to his career of violence. Enraged with a woman who had used offensive language to him, he threw her into a well. Prosecution was com-menced against him; and on the deposition of a great many witnesses, who gave evidence to his furious deportment, he was condemned to perpetual confinement in Bicêtre.'" (P. 14.)

Esquirol and Georget both recognise the same form of disease, and the former considers moral perversion as con-stant and essential in every species of madness. The affir-mation of Esquirol is however much too general, as instances are not wanting in which patients, labouring under mental alienation, have exhibited an amiable, moral disposition, and an undisturbed flow of the natural affections. The ordinary connexion between insanity and moral depravity is neverthe-less striking; indeed the latter is sometimes the earliest in-dication of the former. It is justly remarked by Dr. Haslam, that young persons who exhibit an uncontrollable disposition to vice, notwithstanding the influence of excellent moral pre-cept and example, frequently become deranged in their intellect as they advance in life.* It is equally true that when insanity is hereditary in a family, one member of it may be absolutely deranged, while another, with unimpaired intellect, shall exhibit a frantic violence in his passions, or an inveterate immorality in his habits.

It is an interesting inquiry to the physiologist, how far moral insanity may, in some cases, be connected with a perversion of those *instincts* which man possesses in common with the inferior animals, and which, though they are modified by reason, are in their nature independent of it. Thus a woman loves her child instinctively the moment it is born, before she can have any moral reason for preferring it to the child of another,—she is attached to it by the powerful law of nature, which binds every female animal to its own offspring. The sexual instinct is still more obviously independent of the intellectual and moral nature. These instincts appear, in certain cases, to become perverted, so as to induce forms of moral insanity. Thus a woman is seized with an irresistible impulse to destroy her child, while the perversion of the sexual instinct induces a variety of humiliating phenomena. The consideration of perverted instincts affords a strong point to phrenology, which, regarding all our intellectual and moral powers as instincts, and localising them all in some region or other of the encephalon, refers a disordered state of the intellect, or moral feelings, to disease of the organs on which their manifestations depend;—to this subject however we shall have occasion to recur. There is a form of mental derangement peculiar to old age, which constitutes a variety of moral insanity.

"This disordered state makes its appearance in old men who have never before been insane or suspected of any tendency to mental derangement. It consists, like other forms of moral insanity, in a morbid excitement of passions and a remarkable perversion of the temper and propensities. The whole moral character of the person is changed. 'The pious,' says Dr. Burrows, 'become impious, the content and happy discontented and miserable, the prudent and economical imprudent and ridiculously profuse, the liberal penurious, the sober drunken.' In some elderly persons impulses which had long been effete become of a sudden excited, and a strong tendency to vicious habits is displayed. 'In fact the reverence which age and the conduct suited to it always command, is converted into shame and pity at the perversion of those moral and social qualities which, perhaps, have hitherto adorned the patient's declining days.' This description coincides accurately with the character of moral insanity. There are instances, though rare,

* Observations on Madness, p. 230.

of the appearance of hallucinative madness in old persons, but the case we have now described is of a different character. and consists in a disordered condition of the moral or active powers alone." (P. 25.)

*Monomania.* This is the form of insanity formerly termed *melancholia,* which term is frequently inapplicable, since the hallucination under which the patient labours is sometimes quite the reverse of gloomy. The term *monomania,* first suggested by M. Esquirol, is of more general applicability, and expresses the characteristic feature of the affection.

"This form of insanity," says Dr. Prichard, "is characterised by some particular illusion or erroneous conviction impressed upon the understanding, and giving rise to a partial aberration of judgment. The individual affected is rendered incapable of thinking correctly on subjects connected with the particular illusion, while in other respects he betrays no palpable disorder of mind." (P. 26.)

It is generally believed that the monomaniac is perfectly sane on every subject but that of his own particular hallucination. As far as our experience goes, this is quite the reverse of the fact. We never saw a monomaniac who appeared to us perfectly sane on any one subject whatsoever. In fact, when we consider the constant disposition to recur to the favourite delusion, and the tendency evinced to bring every subject, however remote, to bear upon it, this alone argues so distempered a state of the *associative* faculty—one of the most important in our whole mental constitution—as to preclude the possibility of a healthy exercise of the intellectual powers in general. The fact that the patient may argue with great acuteness on indifferent subjects, is nothing to the purpose, because many patients will argue with equal ability on the very subject on which they are known to be mad. A clever lunatic will often give better reasons for his insane convictions, than many a man in his senses for those tenets which he holds in truth and soberness.

Authors on insanity, however, as on other subjects, are fond of the marvellous; and nothing seems more wonderful, than that a man should be outrageously mad on one subject, and eminently lucid on all others. In our negation of this, we are happy to fortify ourselves by the more weighty testimony of Dr. Prichard.

"The notion, however, which many persons entertain as to the nature of this disease, is far from being correct in its full extent. It is supposed that the mind of the monomaniac is perfectly sound when its faculties are exercised on any subject unconnected with a particular impression, which in itself constitutes the entire disease. Cases are indeed on record, which, if faithfully related, fully come up to this description. In general the real character of monomania is very different. The individual affected is, under ordinary circumstances, calm, and exhibits no symptom of that preturbation and constant excitement which are observed in raving madness. But on careful inquiry it will be found that his mind is in many respects in different condition from that of perfect health. The habits and disposition have, perhaps, been long, in a greater or less degree, in the state which characterises moral insanity. If we advert to the order and connexion of morbid phenomena, we often learn that on a settled and habitual melancholy, or on a morose and sullen misanthropy, long growing and indulged, or on some other disordered and perverted state of the feelings and affections, a particular illusion has more recently supervened. An individual of melancholic temperament, who has long been under the influence of circumstances calculated to impair his health and call into play the morbid tendencies of his constitution, sustains some unexpected misfortune, or is subjected to causes of anxiety; he becomes dejected in spirits, desponds, broods over his feelings till all the prospects of life appear to him dark and comfortless. His inclinations are now so altered that no motive has sufficient influence over him to rouse him to voluntary and cheerful exertion. During this period, if questioned as to the causes of his mental dejection, he will probably assign no particular reason for it. At length his gloom and despondency becoming more and more intense, his imagination fixes upon some particular circumstance of a distressing nature, and this becomes afterwards the focus round which the feelings which harass him concentrate themselves. This circumstance is often some real, occasionally some trifling act of delinquency for which the individual expresses the strongest and perhaps disproportionate self-condemnation. In other instances an unreal phantom suggests itself, in harmony with the prevalent tone of the feelings, which at first haunts the mind as possible, and is at length admitted as reality. Other individuals begin by indulging morose and unfriendly sentiments towards all their acquaintance, magnifying in imagination every trifling neglect into a grievous contumely. They fancy, at length, that they find in some casual occurrence glaring proofs of premeditated designs to ruin them and expose them to the contempt and derision of society. The disease in these cases has its real commencement long before the period when the particular illusion, which is only an accessary symptom, is discovered, and even before it became impressed on the imagination; but it is not until that impression has taken place that the case assumes the proper character of monomania." (P. 27.)

It is justly remarked by our author, that the illusion of the monomaniac usually bears a close relation to his former habits of thought; and that monomania generally supervenes on some disordered state of the moral feelings. In reasoning on insanity, much instruction may be derived from an attentive observation of what passes in our own minds; for the mind of a maniac is composed of the same materials as that of a sane person—but their balance is lost, and the little world of man is no longer "ponderibus librata suis."

Now it must have occurred to every man who has been much occupied with moral reflections, to dwell so long on some one point on which his feelings were deeply interested, that his judgment has begun to lose its ascendancy—the placid light of the intellect has grown dim, and the shadow projected by memory or conscience has broadened and deepened till it has cast its superstitious gloom over his whole moral nature, and he has felt the necessity of receding from the subject as one which his mind was no longer equal to grapple with, and of allowing his faculties to recover their wonted elasticity amid the diversions and occupations of ordinary life. Had he continued to indulge in such contemplations, insanity might have been the result.

Supposing a sane state of mind, there would appear to be this difference in the effect of too long continued attention to subjects of a purely intellectual, and to those of a moral character: in the former instance the mind becomes simply fatigued, and cannot get any further in the inquiry; in the latter there is a tendency to illusion, and the object is magnified or distorted: hence many men go mad of love or religion, for one who takes his start from mathematics or chemistry.

As the illusions of monomania are connected with the peculiar impressions which have been predominant in the mind of the individual, so they must necessarily partake largely of the genius of the age and country in which he lives—its politics—its philosophy—and its superstitions. Thus the Greeks were hunted by furies, the Jews possessed by demons, the Europeans of unenlightened ages bound by the spells of witches, and the men of better times mystified by the dreams of religious or political fanaticism, or tormented by the horrors of ideal pecuniary embarrassment.

It is not always easy to draw the line between hypochondriasis and monomania, since the illusions of the latter state sometimes have reference to bodily ailments.

"A great number and variety of cases coming under the same head, of monomania associated with fear and despondency, are instances in which the apprehensions of the individual are concentrated on his bodily feelings, or relate to some diseased or preternatural state in which he conceives himself to exist. In the first stage this disease is hypochondriasis; the patient dwells constantly on some trifling ailments, which he magnifies in apprehension, broods over, and on account of which he makes himself miserable. In this form hypochondriasis constitutes a variety of moral insanity, and a hundred cases of this description are to be found for one which actually makes the transition into erroneous belief. When this change has taken place, the disorder is accounted a decided one of insanity; as when a man fancies that his head has grown larger than his body, that his legs are made of glass, that he has a wolf or a fish in his stomach. The absurd ideas which the diseased imagination frames, in order to account for trifling bodily suffering, are as numerous as they are strange and surprising. I well remember a lunatic who laboured apparently under neuralgic pains. He fancied that the physician, to whose care he was confided, had the power of torturing him by electricity, and that invisible wires were spread through every part of the house, as conductors of the fluid, which was used at night as the instrument of cruel and tyrannical persecution. Many a flatulent hypochondriac has fancied the existence of a goblin or demon in his stomach; and the association between his internal feelings and the idea by which it was accounted for, has become so firm, that nothing in future has been capable of changing it. A case exemplifying this remark is related by Dr. Jacobi, which is so curious that I have determined to extract it.

" 'A man, confined in the lunatic asylum at Würtzburg, in other respects rational, of quiet, discreet habits, (so that he was employed in the domestic business of the house,) laboured under the impression that there was a person concealed within his belly, with whom he held frequent conversations. He often perceived the absurdity of this idea, and grieved in acknowledging and reflecting that he was under the influence of so groundless a persuasion, but could never get rid of it. It was very curious to observe,' says Jacobi, 'how, when he had an instant before cried, "What nonsense! is it not intolerable to be so deluded?" and while the tears which accompanied these exclamations were yet in his eyes, he again began to talk, apparently with entire conviction, about the whisperings of the person in his belly, who told him that he was to marry a great princess. An attempt was made to cure this man by putting a large blister on his abdomen, and, at the instant when it was dressed, and the vesicated skin snipped, throwing from behind him a dressed-up figure, as if just extracted from his body. The experiment so far succeeded that the patient believed in the performance, and his joy was at first boundless, in the full persuasion that he was cured; but some morbid feeling about the bowels, which he had associated with the insane impression, still continuing, or being again experienced, he took up the idea that another person, similar to the first, was still left within him; and under that persuasion he continued to labour.' " (P. 31.)

A series of cases follows, illustrative of moral insanity and monomania, the relations of these forms of disease, and the transition of one into the other. The earlier cases have, with one exception, been already published by the author in the Cyclopædia of Practical Medicine, we shall therefore pass them over.

A number of similar cases have since been communicated to Dr. Prichard, by several eminent practitioners.

He has received the details of nine cases from Mr. Hitch, the resident medical officer of the Gloucester Lunatic Asylum, who states that he could easily furnish a great number of instances coinciding with Dr. Prichard's description of moral insanity.

Among these is an uncommon example occurring in a child.

"In the spring of 1827 I was requested to visit the daughter of a farmer, in some branches of whose family insanity existed. The little girl was only seven years old. She was reported by her parents to have been a quick, lively child, of ready apprehension, mild disposition, affectionately fond of the members of her family, and capable of quite as much application to her school duties as children usually are. She had been sent home from school in consequence of a great change which had taken place in her conduct. She had become rude, abrupt, vulgar, and perfectly unmanageable; neglecting her school duties, running wildly about the fields and gardens, and making use of the most abusive language, when chidden for her misdemeanours. I found her in this state, with the addition of having become extremely passionate, in consequence of corrections to which she had been subject, and to escape which she was prone to invent falsehoods. She was also changed in her appetite, preferring raw vegetables to her ordinary food; and she would sleep on the cold and wet ground rather than in her ordinary bed. Her parents had no controul over her; indeed she appeared to despise them in proportion as they kindly remonstrated with her. She was cruel to her young sisters; taking every opportunity to pinch or otherwise hurt them, when she thought she could escape observation. She could not apply herself to any thing, but had yet a perfect knowledge of persons and things, and a complete recollection of all that had occurred and of all she had learned previously to her illness. Her general health was much disordered: her little eyes glistered most brilliantly; the pupil was contracted, though expanding widely if she was suddenly excited; the conjunctiva was reddened; the head was hot; the surface of the body of about the natural standard; the extremities of a lower temperature, and the palm of the hand had as completely the peculiar feel of the nervous as a grown person; her person has a disagreeable odour; the bowels were much disordered, from the various strange things she had eaten. Dr. B—— saw her in conjunction with me; and we endeavoured to improve her general health, hoping that by so doing we should remove some exciting cause for her disturbed feelings: we were disappointed. As she grew worse, and her parents by mismanaging, sometimes humouring her, sometimes harshly correcting her, were likely to render her still more disordered, I took her into my own house, and placed her under the care of my wife. At this time she had taken to eat her own fæces, and to drink her urine, and she would swear like a fishwoman, and destroy any thing within her reach; yet she was fully conscious of every thing she did, and generally appeared to know well that she had done wrong. Having committed some mischief, or destroyed something which was fragile, she would often run to my wife, and exclaim, " Well! Mrs. H——, I have done it! I have done it! I know you will be angry, but I can't help it. I felt I must break it, and I could not let it alone until I had!" Amongst her pleasures was that of dirtying herself as frequently as she had clean clothes: indeed, she would rarely pass her excretions at the proper place, but reserved them for the carpet of the sitting-room, or for her own clothes. When she had accomplished this end, she would jump about and exult; but the little creature would often induce my wife to smile at her, when, with an expression of countenance, (which was always intelligent,) made up of cunning, feigned regret, and a subdued smile, she would say, "Well, Mrs. H——, 'tis too bad of me; 'tis really very foolish, and I will try to be better; but you must forgive me, because I am mad." At other times she would be so far conscious of her situation as to cry bitterly, and express her fears that she should become like her aunt, who was a maniac. In addition to all these indications she lied, stole any thing which she thought would be cared for, and either hid or destroyed it; and swore in language which it is difficult to imagine that the child could ever have heard.

" ' I could never detect in her any fixed idea, either of fear or belief, which influenced her conduct. She acted from the impulse of her feelings, and these were unnatural and unhealthy.

" ' She recovered in about two months.' " (P. 55.)

*Mania.* Our author is not diffuse on the phenomena of mania, or raving madness, nor is it necessary to be so. The utter subversion of the intellectual and moral powers, which constitutes this affection, defies classification; all the faculties are over excited without any permanent object on which to exercise themselves. Dr. Prichard gives, as a general illustration of this form of disease in its greatest intensity, an abstract of the description by Chiaruggi, which is cited with high commendation by Professor Heinroth.

" Among the phenomena of the first stage of this disease, we are at first struck by impetuous, audacious, shameless habits, a bold menacing aspect; the natural evacuations are deficient; the skin becomes of a slaty colour; the forehead contracted; the eyebrows drawn up; the hair bristled; the breathing hurried. The countenance begins to glow; the eyes become fiery and sparkling; the looks are wandering, and scarcely ever fixed; the eyelids are by turns drawn widely open, and closely shut; the eyeballs are prominent, as if pushed forward out of the orbits. With this wild and menacing appearance is combined a patient endurance of hunger, and a remarkable insensibility of cold. If sleep visits the patient at all, it is short, unquiet, and easily disturbed. In the second stage, anger, violence, and the loss of reason, manifest themselves in their greatest intensity; shrieking, roaring, raging, abusive expressions and conduct towards the dearest friends and the nearest relations, who are now looked upon as the bitterest enemies. The patient tears his clothes to tatters, destroys, breaks in pieces whatever comes in his way. A striking and characteristic circumstance is the propensity to go quite naked. Whoever touches the patient is abused or struck by him. Strange confused ideas, absurd prejudices, occupy the mind. Stillness soon follows, or a murmuring sound, as if the patient were alone: on the other hand, when he is alone, talking and gesticulating as if he were in company. If such individuals are confined and tied during the height of their paroxysms, for their own security or that of others, nothing can be compared to the truly satanical expression which their countenances display. In this state they throw hastily away, with cries and shrieks, all the food presented to them, except fluids, which thirst compels them to receive. When, after some days, hunger begins to be felt, they swallow every thing with brutal greediness; they even devour, as it has often been observed, their own excrements, which, black and offensive, escape from them in great quantity, or smear with them clothes, beds, and walls. Notwithstanding his constant exertion of mind and body, the muscular strength of the patient seems daily to increase; he is able to break the strongest bonds, and even chains; his limbs seem to acquire a remarkable nimbleness and pliability, and a singular aptitude of performing movements and actions which appear almost supernatural. Chiaruggi saw a woman, who, clothed in a strait-waistcoat, and laced down in her bed like a child in a cradle, drew out her limbs from this double confinement, with the greatest nimbleness and pliancy. Bold, however, and impudent as such patients are, yet they are, according to common observation, although not without exceptions, easily daunted by a strong threatening voice, by the sight of stocks, by close though harmless restraint. After their violence has expended itself, they become still, gloomy, appear to be reflecting or brooding over something; but they break out again, before it can be anticipated, into a new storm of rage. At length comes on the *third stage*. A real cessation of violent paroxysms now ensues, exhaustion, sleep, though unquiet, disturbed by fearful dreams. The pulse is small, the aspect of the whole body squalid, the countenance pallid and meagre. The patient is obdurately silent, or sings and laughs in a strange manner, or chatters with incessant volubility. These uncertain intervals, which often put on the appearance of fatuity, are frequently interrupted by new but short renewals of violence. Memory for the most part remains unimpaired through all the stages, and during the highest intensity of the disease the senses appear to acquire an unusual degree of acuteness and susceptibility. A patient who had recovered described to Chiaruggi all the scenes of his wild reverie and long-continued mental preturbation. It has often been observed that maniacal patients of this description are never attacked by any epidemic, and are seldom affected by any contagious malady. According to Mead and many others, even consumptive disorders, dropsies, and other chronic maladies have disappeared on the accession of violent insanity. When patients are not freed from the disease after a succession of attacks, which come on like so many paxoxysms of fever, one or the other of the following events ensues: either the powers of mind are exhausted to that degree that the disease subsides into a permanent fatuity; or this appearance of fatuity is only a space of calmness interposed between relapses of violent madness, which now and then break out, like the eruptions of a volcano after a long period of repose; or the patient falls into a state of melancholy or of complete mental confusion; or, finally, his madness becomes chronical, and he scarcely recovers from this condition, in which sense and understanding appear to be lost in incoherence. Chiaruggi saw a woman who had sat during twenty-five years on a stone-floor, in a fearfully demented state, beating the ground with her chains without ceasing by day or by night." (P. 76.)

No particular examples are given. Cases of furious mania serve only to cover paper, and illustrate nothing in philosophy or medicine. It is often observed that violent mania soon arrives at a kind of imperfect crisis, the intensity of the symptoms abates, and the disease passes into a chronic state. M. Esquirol thinks that this change generally, or at least frequently, takes place within a month from the first accession of an acute attack.

*Protracted Insanity.* Our author observes that the ultimate tendency of insanity, is to produce a state of mental decay or *dementia.* The transition to this however is gradual, consisting of a stage of protracted mental derangement, in which the majority of patients in lunatic asylums are found to exist for years, and which sometimes continues during life, without ever terminating in complete dementia. This state is a combination of mania, or monomania, and dementia, in which the symptoms of these different forms of insanity are not distinctly marked, but in some measure blend and pass into each other.

"The symptoms belonging to one form of madness are often confounded with those of another, and from the combination there results, as it has been remarked by a writer of extensive observation and research, an assemblage of phenomena which bears little or no similitude to the description found in books and commonly entertained." (P. 80.)

Dr. Prichard animadverts with justice on the inaccuracy of the opinion, that in all cases of insanity there are successive periods of monomania, mania, and dementia. Insanity is a subject, of all others, on which it is easy to write imposingly, without a very large store of facts; a little talent for classification, and a little poetry of style, may give some currency to a work got up entirely in the closet.

*Incoherence or Dementia.* This is a state in which unconnected and imperfectly defined ideas chace each other rapidly through the mind; the powers of continued attention and reflection are lost, and even the perceptive faculty at length becomes indistinct. The term *demence* or *dementia* was first applied to this form of disease by Pinel. A similar term has long been vernacular in Scotland: when a man is conceived to have lost his wits, they say he is *demented.* Dr. Prichard, preferring native to exotic terms, proposes to call this state *incoherence,* which designation however is perhaps rather too indefinite to be applicable to any specific case of insanity, since many persons possessed of sufficiently good understanding, are prevented from exercising it by the volatility arising from defective education and absurd habits; and we fear that if *incoherence* is to be considered as a form of insanity, a great many of the fair sex, and not a few of the other, are in danger of finding their way into an asylum. Pinel has defined dementia to consist in "ideas incoherent among themselves, and bearing no relation to external objects." This definition he expands in terms which Dr. Prichard gives in the original, as being difficult to translate with precision. This difficulty, we conceive, arises from a want of precision in the original itself; the French are generally bad metaphysicians, and use the language of psychology in too lax an acceptation; we shall, however, attempt to give the English of the passage in question.

"A turbulent and incontrollable mobility, a rapid, and, as it were, instantaneous succession of ideas, which appear to rise and germinate in the understanding, without any impression made on the senses; a continual and absurd flux and reflux of chimerical objects, which alternate with each other, embarrass and destroy each other without intermission, and without any relation subsisting among them; the same incoherent but placid concourse of moral affections, of the sentiments of joy, sorrow, and anger, which, arising fortuitously, disappear without leaving a trace behind, and without having any correspondence with the impression of external objects: such is the fundamental character of the dementia of which I speak." There is more sound than sense in all this; obscurity of thought is veiled by cloudiness of diction.

"Incoherence is either a primary disease, arising immediately from the agency of exciting causes on a constitution previously healthy, or it is a secondary affection, the result of other disorders of the brain and nervous system, which, by their long duration or severity, give rise to disease in the structure of those organs. The causes which produce the state of incoherence as an original disorder are nearly the same with those which in other cases excite madness in the first instance; they are such agents as break down the powers of the mind by their overwhelming influence, or destroy them by vehement emotions. Secondary incoherence or dementia follows long-protracted mania, attacks of apoplexy, epilepsy or paralysis, or fevers attended with severe delirium. This decay of the faculties has been termed fatuity or imbecility, and it has been confounded with idiotism, which in all its degrees and modifications is a very different state. The distinction, which is very important, has not always been kept in view by writers on disorders of the mind, and even in the works of Pinel we find it sometimes overlooked. M. Esquirol has the merit of having drawn more accurately the line of discrimination. He refers to dementia all the cases of effete or obliterated intellect which are the results of maniacal or other diseases, and are incident to persons originally possessed of sound faculties, and includes those defects only under idiotism, which are original or congenital. 'The imbecile,' he observes, 'have never possessed the faculties of the understanding in a state sufficiently developed for the display of reason. The victim of dementia was once endowed with them, but has lost this possession. The former lives neither in the past nor the future; the latter has some thoughts of times past, reminiscences which excite in him occasional gleams of hope. Imbecile persons in their habits and manner of existence display the semblance of childhood; the conduct, the acts of the demented preserve the characteristics of consistent age, and bear the impress derived from the anterior state of the individual. Idiots and cretins have never possessed memory, judgment, sentiments; scarcely do they present, in some instances, indications of the animal instincts, and their external conformation plainly indicates that they were not organized to be capable of thought.'" (P. 85.)

The following table, extracted from Esquirol's treatise, exhibits the comparative influence of different causes in the production of *dementia*. The first column refers to patients admitted at the Salpêtrière in 1811-12; the second to those treated in M. Esquirol's private establishment, who were persons among the higher or more opulent classes of society.

| *Physical Causes.* | Number of individuals. | | Total. |
|---|---|---|---|
| Disorders connected with the catamenia | 11 | 4 | |
| Critical period | 29 | 6 | |
| Consequences of childbirth | 5 | 3 | |
| Blows upon the head | 3 | 0 | |
| Progress of age | 46 | 3 | |
| Ataxic fever | 1 | 2 | |
| Suppression of hemorrhoids | 0 | 2 | |
| Mania | 14 | 4 | 195 |
| Melancholia | 13 | 2 | |
| Paralysis | 3 | 2 | |
| Apoplexy | 3 | 2 | |
| Syphilis and abuse of mercury | 6 | 8 | |
| Faults of regimen | 0 | 6 | |
| Intemperance | 9 | 6 | |
| Masturbation | 4 | 7 | |
| *Moral Causes.* | | | |
| Disappointed love | 1 | 4 | |
| Fright | 4 | 3 | |
| Political excitement | 0 | 8 | 40 |
| Disappointed ambition | 0 | 3 | |
| Poverty | 5 | 0 | |
| Domestic griefs | 8 | 4 | |
| | 192 | 73 | 235 |

Dr. Prichard describes the phenomena of dementia as referable to four distinct stages:

1. That of *impaired memory*, which obtains especially with regard to recent events.

2. That of *irrationality*—a total loss of reasoning power, arising from the absence of voluntary control over the thoughts.

3. That of *incomprehension*, in which the patient cannot understand anything that is said to him.

4. That of *inappetency, or loss of instinct and volition*, in which the individual loses even his animal instincts, and does not obey the calls of nature.

He observes, that dementia approaches most gradually when it occurs as the accompaniment of old age, and that its several degrees are most distinctly recognised in such instances.

*General Paralysis complicated with Insanity.* There is a peculiar modification of paralysis which often accompanies the advanced stages of insanity, and especially those cases which are passing, or have already degenerated into dementia. "M. Esquirol was the first writer who directed the attention of physicians to this morbid state, and pointed out the incurable nature of insanity complicated with paralysis."

This form of paralysis is much more frequent in men than in women. Of 109 insane paralytic patients, who came under the care of M. Esquirol, at Charenton, during three years, 95 were males. The same author made similar observations at the Bicêtre, and the fact has been corroborated by Dr. Foville, chief physician to the asylum at St. Yon, near Rouen. Of 334 lunatics at that institution, 31 were paralytic, and of these 22 were men. At Charenton, M. Esquirol has found the proportion of paralytics still greater, forming no less than one sixth of the whole number of patients; of 609 cases of insanity, 109 were also paralytic. The proportion of male to female cases is immense: thus of 366 male lunatics, 95 were paralytic; of 253 females, only 14.

M. Calmeil has marked three degrees in the general paralysis of the insane. The first degree is characterised merely by an impediment in the motions of the tongue, which renders the articulation imperfect, the patient drawling and stammering, like an intoxicated person; the tongue however can be naturally protruded, and the muscles of the mouth and face preserve their usual position. In the second degree the defect of articulation is greater, and the patient is incapable of uttering a word distinctly. The limbs are now affected with paralysis; the patient rises from his chair with difficulty, by fixing his hands upon its arms, and when he attempts to walk, his gait is tottering. When in the recumbent posture, he moves all his limbs with freedom. The upper extremities are less affected than the lower, yet in some instances the arms are impeded in their movements, and raised to the head with difficulty. The chin has a tendency to fall upon the chest, from the relaxation of the muscles of the neck, and the trunk is ill poised upon the pelvis, from the impaired action of its muscles. The senses in general are much obtunded; the patient however still continues to feel, see, hear, and smell. The excretions escape without notice, whether from paralysis of the sphincters, or the inattention of the patient—more probably the latter. Patients affected with this degree of paralysis, are generally in an advanced stage of dementia. In the third degree, the patient is nearly reduced to a state of vegetation. Articulation is almost or entirely lost. The patient cannot stand, and at last loses all power of moving the lower extremities; the hand and arms are less completely paralysed. Frequently no trace of intelligence remains; the patient waits till food is put into his mouth; he is insensible to the calls of nature, and though he still retains the senses of sight and taste, and perceives strong odours, he pays no attention to surrounding objects. In the first and second stages just described, the functions of organic life are nearly in a healthy state; the circulation is natural, sleep undisturbed, excretion is duly performed, and the flesh remains firm and plump. In the third stage however digestion is impaired, and the frame becomes emaciated. At length the integuments slough on those parts most subjected to pressure; œdema of the most depending parts supervenes, and hectic fever closes the scene. Death seldom occurs as the simple consequence of the cerebral disease, the abdominal or thoracic viscera being generally affected.

Dissection has proved, that this species of paralysis is almost uniformly connected with chronic inflammation of the substance of the brain, especially near its surface. The observations of M. Calmeil on this subject will be presently alluded to. From numerous observations of M. M. Calmeil, Esquirol, and others, it results that this kind of paralysis commences in some instances a long time after the accession of insanity, in others simultaneously with it, while in a few it precedes it. M. Calmeil states, that at Charenton the paralysis generally supervened soon after the commencement of insanity. A few cases have occurred to M. Esquirol, in which patients seized with general paralysis have retained perfect vigour of intellect for some time, but have subsequently fallen into a state of mental derangement. The mean duration of the paralysis of the insane, is stated by Calmeil at thirteen months; and he confirms the observation of Esquirol, that few patients survive longer than three years. The disorder is generally progressive, though a few individuals have suddenly recovered so far as to be able to walk several miles a day, and to resume, for a time, their ordinary occupations. The prognosis, however, is on the whole most unfavourable. M. Royer Collard, during twenty years' experience in a very large establishment, did not witness a single recovery. Esquirol and Calmeil agree in considering the malady as generally incurable; the former however has seen three, and the latter two, instances of recovery.

The eighth section of this chapter is on the state of the sensorial and intellectual functions, in cases of insanity. This is a subject which belongs quite as much to metaphysics as to medicine. It is moreover one whose successful exposition would require a more extended acquaintance with the philosophy of the mind, than has usually been evinced by medical writers; and a more exact agreement in the nomenclature and classification of the intellectual powers, than has hitherto obtained among psychologists, or is perhaps attainable in so abstract a science. We shall therefore content ourselves with recommending this section to perusal, as characterised by the same good sense and information which are conspicuous in other parts of the work.

The ninth section is on *Disorders of the Physical Functions attendant upon Madness.* The phenomena proper to insanity are those connected with the disordered state of the mental faculties; to these however is superadded in many, perhaps in most, cases, more or less disturbance of the organic functions, as digestion, secretion, and excretion. Pinel is of opinion, that the primary disorder in cases of insanity, is seated in the stomach and intestines, and that the brain is sympathetically affected. Others have regarded the disease of the brain as primary, and the disorder of other parts as contingent upon it. Whichever of these views be entertained, and, in the present state of our knowledge, it would be extremely rash to adopt either with confidence, it is certain that derangement of the organic functions is very frequently coexistent with mental alienation. In mental disorders of slow accession, and especially in melancholia, the functions of the systems are generally torpid; the circulation is languid, the pulse feeble and slow, the skin cold and clammy, the bowels constipated, often in an extreme degree, the appetite bad, the digestion impaired, and, as a necessary consequence, the frame emaciated, and the strength diminished. In violent mania, on the other hand, symptoms of febrile excitement very generally prevail, and especially those indicative of increased determination to the head. The latter undergo occasional exacerbations during the progress of the disease: violent pulsation of the carotids, increased heat of the scalp, redness of the eyes, loss of sleep, and irritability of temper, precede or accompany each severe renewal of the maniacal symptoms.

The third chapter is on the terminations of insanity.

Insanity is usually considered as having three terminations: *recovery*, *dementia*, and *death*. The termination in dementia has already been mentioned.

*Recovery* is the event in a large proportion of cases, but the probability of a fortunate issue is influenced by the following circumstances.

*The complication of insanity with other cerebral disorders.* The most unfavourable complication is that of general paralysis; if this affection be present, even in the slightest degree, the case must generally be considered hopeless. Epilepsy is also a very unfavourable complication; it is much less so however in those cases where the fits occur only at periods of high maniacal excitement, as the effects of a transient cause, than in those where violent mania appears as the sequel or occasional interlude of inveterate epilepsy, which latter case is a very desperate one.

*The form of the disease.* This is of great importance with reference to the event. The following comparative statement of recoveries among the patients at Charenton, is given by M. Esquirol.

| | | | |
|---|---|---|---|
| Mania . . | 115 | cases out of | 226 |
| Monomania | 91 | . . . . | 289 |
| Dementia . | 3 | . . . . | 99 |
| | 209 | | 614 |

In the total number of cases are included 109 paralytics, 19 epileptics, and 4 idiots; so that the number of curable cases may be considered as reduced to 487, of which number more than two-fifths were restored. "It is remarkable," observes Dr. Prichard, "that the greater sanability of maniacs, in comparison with monomaniacs, had place only in males. From this it would appear, that monomania is comparatively a more curable disease in females than in men."

*The period of the disease.* This is a very important consideration; the chance of recovery being much greater when the case comes early under treatment. In the practice of Dr. Willis, nine out of ten are stated to have recovered when placed under his care within three months after the first attack. Under similar circumstances, Dr. Finch treated successfully 61 cases out of 69. Dr. Burrows cured 221 out of 242 recent cases; and the practice of the Retreat, near York, affords a proportion of at least seven recoveries out of eight recent cases.

A table of recoveries at the Gloucester Lunatic Asylum is given on the authority of Mr. Hitch, the resident medical officer, which affords similar results, and contains some examples of restoration to reason after so long a duration of insanity, as to show that we ought never absolutely to despair.

*Age.* M. Esquirol has found that the most favourable age for recovery is between the twentieth and thirtieth year, and that few are cured after the age of fifty. From the table of recoveries at Charenton, it appears that the greatest number of cures were in patients from twenty-five to thirty, and from thirty to thirty-five years of age. The proportion of recoveries diminishes progressively from the forty-fifth year onwards. The diminution is more abrupt in women, and more uniform in men. Cases occurring before the twentieth year are most numerous in males. In men the disease most frequently makes its invasion from twenty to twenty-five, and from twenty-five to thirty; in women between twenty-five and thirty. The experience of Charenton shows that advanced age does not preclude hope, twenty men having recovered after their fiftieth year; in which number are included four out of twelve lunatics who were more than seventy years old.

*Sex.* Insanity has generally been observed to be more curable in women than in men.

*Season*, and the *circumstances of the constitution*, are alluded to by our author as modifying the prognosis; these however we need not dilate upon.

The general proportion of recoveries in cases of all kinds is very differently estimated by different writers. The experience of Dr. Burrows affords 240 cures in an aggregate of 296 cases of various descriptions—221 in 242 recent cases, 19 in 64 old cases—giving an average of 81 in 100 cases of all sorts, and 91 in 100 recent cases.

Dr. Jacobi ascribes the large proportion of recoveries in England to the practice which he believes to obtain in this country, of discharging patients from the asylums too soon after apparent restoration. In this gentleman's establishment at Sieburg, in Westphalia, 40 out of 100 cases were dismissed completely cured, and 6 with alleviation of symptoms. Dr. Prichard justly remarks, that Jacobi is certainly mistaken in supposing that patients are dismissed too early from lunatic asylums in England. "It is plainly not the interest of those who conduct private establishments to err on this side; and in most of the public hospitals in this country, the proportion of recoveries is by no means so great as to require such an explanation."

M. Esquirol has for many years been engaged in extensive researches into the statistics of lunatic asylums. In the Dict. des Sciences Med., he gives the following table of recoveries, taken from the reports of the French establishments.

| *French Lunatic Asylums.* | *Dates.* | *Admissions.* | *Recoveries.* |
|---|---|---|---|
| Charenton .. | from Nov. 22, 1798 to 1800, 22 July, | 97 ... | 33 |
| | 1803 . . . . . . | 499 ... | 161 |
| Salpêtrière ... | from 1801 to 1805 . . | 1002 ... | 407 |
| — ... | from 1804 to 1813 . . | 2005 ... | 1218 |
| — ... | from 1806 to 1807 . . | 531 ... | 286 |
| — .. | from 1812 to 1814 . . | 891 ... | 413 |
| In M. Esquirol's private establ. | from 1801 to 1813 . . | 335 ... | 173 |
| | Totals . . | 5360 | 2691 |

In a still larger calculation, contained in the Mem. Acad. Roy. Med., 4968 cures are reported out of 12,592 cases, treated at the Salpêtrière and Bicêtre. A more recent report of the same eminent observer, referring to the practice of Charenton in 1826-28, gives a proportion of recoveries amounting to one third of cases of all descriptions; and an official report, made at Paris in 1825, of the state of lunatic hospitals for the three preceding years, affords nearly a corresponding result; the recoveries being 34 in 100, including cases deemed curable and incurable. Esquirol has collected the following statements from English lunatic hospitals.

| *English Lunatic Asylums.* | *Dates.* | *Admissions.* | *Recoveries.* |
|---|---|---|---|
| In Bethlem Hospital . | from 1748 to 1794 . . . . | 8874 ... | 2557 |
| — — . | in 1813 . . . . | 422 ... | 204 |
| In St. Luke's . . . | from 1751 to 1801 . . . . | 6458 ... | 2811 |
| In York Asylum . . . . . . . . . . . . . . . . . | | 599 ... | 286 |
| In the Retreat near York, | from 1801 to 1814 . . . . | 163 .. | 60 |
| | Totals . . | 16516 ... | 5918 |

" From these calculations," observes Dr. Prichard, " it appears that the proportion of cures formerly obtained in English lunatic asylums was in some instances less than that which is reported from the celebrated hospitals in France. This is the more remarkable when we take into our account the peculiar regulations of the great lunatic establishments of London. The hospitals of Bethlem and St. Luke impose certain exclusions, elsewhere unknown. They reject all patients who have been more than twelve months insane; those affected by paralysis, however slight, and by epilepsy or convulsive fits; idiots, the aged and infirm; those discharged uncured from other hospitals: there are likewise other exclusions besides those above mentioned, and all persons who have not recovered at the expiration of one year are dismissed. Yet, on comparing the reports of these hospitals with those of other institutions, the regulations of which are less favourable to a high proportion of cures, and where no selection or exclusion exists, we do not find, as Dr. Burrows remarks, the relative number of recoveries to be so great as might be expected. It is indeed surprising to observe that the reports of Bethlem Hospital, of a century and a half ago, give a greater proportion of cures than those of many years preceding 1817, when an improvement took place in the arrangements of that establishment. Dr. Burrows remarks on the authority of Stow, who derived his information from Dr. Tyson, physician to Bethlem Hospital, that 'from 1684 to 1703, 1294 patients were admitted, of whom 890 were cured, which is a proportion of two in three. But from 1784 to 1794, 1664 patients were admitted, of whom 574, or rather more than one in three, recovered.'" (P. 139.)

So great a difference could not be accounted for by any presumed inferiority of skill in the practitioners, or of arrangement in the institutions of this country; nor is there any reason to believe that any such inferiority existed. Since the removal of Bethlem Hospital to a new site, the average of recoveries has greatly increased. From a table supplied by Mr. Lawrence, it appears that, out of 2060 cases, admitted between 1819 and 1833, 1124, or considerably more than one half, were cured. The report of the Stafford Asylum, from 1818 to 1828, gives about 43 cures in every 100 cases; that of Wakefield County Asylum, from 1819 to 1826, about 42 in 100. A report of the Lancaster County Asylum, obtained by our author through Dr. Whalley, states a proportion of recoveries amounting to about 40 in 100 of the total number of cases (1750,) from 1817 to 1832. A report from the Gloucester Asylum, drawn up by Mr. Hitch, and referring to the years 1823-32, gives a very favourable list of recoveries,—234 out of 516 cases; without taking into account a considerable number of recoveries, which afterwards took place among those dismissed as relieved and on trial. In this establishment there is no selection of cases, lunatics of all descriptions being freely admitted. Our author concludes his very ample and excellent view of this important subject, with a table obtained from Mr. Tuke, of York, from which it appears that, out of 334 patients, admitted into the Retreat in that city, from 1812 to 1833, 168 have recovered, 50 have died, 37 have derived no benefit and been removed, 10 have left the asylum in an improved state, and 69 remain.

*Termination in Death.* Insanity is not, on the whole, a disease very dangerous to life; the disturbed state of the brain having reference rather to its operations as the organ of the mind, than to its general functions as a part of the corporeal frame. Many lunatics have remained in asylums for thirty, forty, or fifty years; nor are cases of unusual longevity wanting among the insane. Still, however, the brain is in a morbid state, and hence very liable to inflammation and congestion, with their ordinary results, as apoplexy, palsy, and convulsive affections, to which the insane are much more liable than other persons. Another mode in which madness terminates fatally, is by the exhaustion arising from continued excitement; the patient, continually restless, agitated, and sleepless, dies, worn out and emaciated. The symptoms of high excitement are most prevalent at the commencement of insanity; hence the mortality is greatest during the first two years, and especially the first. Esquirol states that, of 790 deaths in the Salpêtrière, between the years 1804 and 1814, 382 happened within the first year after admission, 227 in the second year, and 181 during the seven succeeding years. Many insane patients die of diseases of the abdominal and thoracic viscera. Such complications of insanity are too frequent to be regarded as accidental, although it may puzzle pathologists to account for them. We should be much disposed to regard defective innervation as a frequent cause of their occurrence; it being established, by the experiments of Dr. Wilson Philip, that the abstraction of the nervous energy from a part gives rise to disordered action of its minute vessels. The coexistence of phthisis with insanity is so frequent as to have been made the subject of particular observation. M. Esquirol states, that phthisis often precedes or accompanies the invasion of melancholia. The patient loses his strength, and becomes emaciated; he has slow fever, sometimes accompanied with cough and diarrhœa, while the symptoms of insanity are rather increased than diminished, and continue until death. On dissection, the lungs are found to be tuberculated, or affected with melanosis. Dr. Greding found that 40 out of 100 maniacs, and 20 out of 24 melancholics, laboured under phthisis: according to the same writer, 76 out of 100 maniacs, and 20 out of 24 melancholic patients, had effusion in one or both cavities of the pleura.

Diseases of the heart are not uncommon. M. Foville found some organic change in the heart or great vessels, in five out of six of all the bodies of lunatics which he examined after death, during three years: the most frequent morbid state was hypertrophy. Dr. Prichard concurs in the opinion of M. Foville, that these lesions are more frequently results of the continued violence done to the thoracic organs by the agitation, efforts, and cries of the patient, than predisposing causes of cerebral disease.

Diseases of the intestinal canal are frequent among the immediate causes of death. A protracted state of obstinate constipation is at length succeeded by, or alternates with, diarrhœa, which terminates in fatal dysentery. On examination after death, the intestines are found loaded with indurated matter, or empty and pale, with discoloured patches, abrasions, ulcerations, or gangrenous spots in the mucous coat.

In the advanced stage of insanity, cachectic disorders are prevalent; as scaly or papular eruptions, scorbutic affections of the gums, furunculi, and a disposition to sloughing in different parts of the body. These are accompanied by diarrhœa and griping pains, and the patient dies in a state of extreme emaciation.

Such are the more important of those causes of death which seem to have some peculiar relation to the state of insanity. There are some others to which, though they may be considered as accidental, the physical condition of insane patients renders them particularly obnoxious. Among these are pulmonary catarrh and typhoid fever: cephalic fevers are, as might be expected, peculiarly fatal to lunatics. The diagnosis of accidental diseases in the insane is attended with much difficulty, since they have a great tendency to make unfounded complaints, and either conceal or are insensible to the presence of real disease. Patients should therefore be carefully watched; and altered habits, and the expression of dejection and suffering in the countenance of a lunatic, should lead to an immediate examination of his physical state, as they probably indicate the approach of some serious malady.

The great mortality in cases of dementia is accounted for by M. Esquirol, on the ground that dementia is the ultimate condition to which all continued insanity leads, and the condition also which is most frequently complicated with paralysis. A much greater mortality has been observed among insane men than women, both at Charenton and in several English hospitals: this may be partly ascribed to the greater prevalence of paralysis in men. With respect to season, M. Esquirol has observed that December, January, and February, are the most fatal months to insane persons.

The fifth section of this chapter is on relapses and recurrent insanity. In many instances, the recovery from madness is complete; in others, and perhaps the majority, the disease is curable only to a certain point, and the patient retains such a susceptibility to it, that, although he may be perfectly rational, a studious avoidance of all sources of mental inquietude is necessary to keep him so; and, even when all tendency to insanity appears to have passed away, the fact of its having once existed affords ground for fearing its recurrence; an observation which is true with reference to all the diseases of the nervous system.

The fourth chapter treats of the causes of insanity.

The predisposing causes are considered under the heads of constitutional predisposition, whether hereditary or original; of sex; of age; of temperament; of previous attacks of insanity, and other diseases of the brain; of education. These subjects are treated by our author with his usual judgment: several of them, however, have been already incidentally alluded to under other heads, and, on the whole, they are not of a nature susceptible of much new illustration. We therefore pass on to the third section, on the

*Productive causes of insanity.* These are divided into *moral* and *physical;* and the influence of these will be most accurately estimated by a reference to the comparative tables given by Dr. Prichard.

In the fourth section, the moral causes of insanity are ably commented on. Some important calculations are stated in reference to the comparative frequency of insanity among married persons, the results of which are highly favourable to the holy state.

The fifth section is on the physical causes of insanity. Those treated of by our author are, injuries of the head, insolation and exposure to heat, metastasis, intoxication, sensuality, intestinal irritation, morbid states of the uterine system. Under the head of intoxication, it is remarked that, among 336 lunatics in M. Esquirol's establishment, only three appeared to have lost their reason through this kind of intemperance; while, in the public asylums in England, it is well known that, in a large proportion of cases, dram-drinking has been the exciting cause.

The fifth chapter is on the *Morbid Anatomy of Insanity*. We have only space for some of the observations on lesions detected in the brain. Those of other organs, though properly noticed in so complete a treatise as Dr. Prichard's, are of minor interest, as being less uniform in their occurrence, and less essentially connected with insanity. The most important observations are those of

*Greding*, who has recorded the following facts:

"1. In the *cranium*. 'Experience has proved,' as he says, 'that the skulls of almost all insane persons have a natural shape.' Of sixteen cases only of the whole number examined, viz. nearly 220, the forehead was contracted, the temples compressed, and the occiput large and expanded. In a few the head was elongated and compressed at the temples. Some had a head almost round, or of a square shape: these were epileptic idiots. Two had small heads, quite circular: these were epileptic madmen. Of 216 cases, including those of madmen, idiots, and epileptics, the skull was unusually thick in 167: this fact was observed in 78 out of 100 cases of raving madness, and in 22 among 30 of idiotism. In many cases the cranium was remarkably thin. Holes were observed in the inner table in 115 out of 216 cases: in other instances bony projections from the inner surface.

"2. *Membranes*. Dura mater firmly adherent to the skull in 107 out of 216 cases; in a few instances of a blueish black colour, thickened and partially ossified. Pia mater thickened and opaque more or less in 86 out of 100 cases of mania; beset with small, spongy bodies in 92 out of 100: these bodies were often united to the surface of the brain, and were in some instances the seats of ossific deposits.

"3. *Brain*. Cerebral substance softer than usual in 118 out of 216 cases: soft and pulpy in 51 cases of mania out of 100, as likewise in 19 out of 24 cases of melancholia, in 8 out of 20 epileptics, and in 16 out of 30 idiots. Those maniacs who had the cerebrum softened had the cerebellum still more soft and pulpy.

"4. *Effusions*. Between the dura and pia mater in 120 out of 216 cases; in 58 out of 100 maniacs. Between the pia mater and the surface of the brain in 28 among 100 maniacs. Lateral ventricles in 29 very full of serum, in 23 ready to burst; in 10 among 24 melancholics astonishingly distended. Third ventricle quite full in 57 of 100 maniacs, and in 16 of 24 melancholics. Fourth ventricle ready to burst in 80 out of 100 maniacs, and quite empty in only 3: completely distended in every one of 24 melancholics.

"*Other appearances*. Plexus choroides in a nearly healthy state in only 16 out of 216 cases, thickened and full of hydatids in

96 out of 100 maniacs. Lateral ventricles either larger or smaller than natural in many cases. Softness of parts of the brain, as of the tubercula quadrigemina in some cases." (P. 210.)

*Haslam*, whose observations are deservedly familiar to the profession in this country: we shall therefore not quote them.

*Georget*, who has noticed the following appearances:

"Irregular conformations of the cranium, the prominences of which are developed irregularly, those of the right side being generally larger than those of the left; some skulls having the lateral diameter of equal extent with the antero-posterior, and the cavities of the base irregular in extent; some skulls, one in twenty, thickened partially or generally; more frequently the bones hard, white, without diploë, resembling ivory; some very light. Dura mater rarely changed; sometimes adherent to the skull, thickened, containing deposits of bone. Arachnoid displaying in places additional laminæ of a red or grey colour; sometimes thickened but smooth. Pia mater injected; or thickened and infiltrated with serum, giving at first the appearance of a gelatinous deposit. Volume of the brain sometimes less than the cavity of the cranium seems to require. Some brains very hard, cut with difficulty; the white substance glutinous, elastic, and suffering distention; more frequently the brain is soft, the grey matter being pale and yellowish, and the white substance discoloured, of a dirty white, the colour and consistence of these portions almost confounded. The convolutions separated by serosity, and the pia mater thickened. Interior cavities of the brain appearing in some instances very large, in others small, often filled with a serous fluid remarkably clear and limpid; plexus choroides exsangueous, containing hydatiform vesicles. Partial softenings of the brain; erosions, ulcerations of the surface of the ventricles. Cerebellum generally softer than the cerebrum; sometimes partially softened. Mesocephalon, medulla oblongata, and medulla spinalis rarely displaying morbid changes of structure."

*Bayle*, who, after extensive observations at Charenton, comes to the conclusion that the proximate cause of insanity is seated, not in the substance of the brain, but in its membranes, from the inflammation of which effusions follow, producing the symptoms of different degrees of compression of the brain, corresponding accurately with those of insanity in its progressive stages. With respect to M. Bayle's *nouvelle doctrine*, we have only to remark, that his anatomical observations differ from those of all other inquirers, and that his pathological views are altogether at variance with facts. The progressive symptoms of insanity are not those of gradually increasing compression of the brain: if they were so, a case of chronic hydrocephalus would be one of insanity; instead of which, it is well known that children affected with the former are usually particularly acute and intelligent.

*Calmeil*, who, labouring in the same field as the last-mentioned writer, has made his observations exclusively on the *general paralysis* of the insane; and most of the phenomena noticed by him agree with those which other morbid anatomists have found in connexion with mental derangement, whether complicated with paralysis or not.

The consistence of the white substance is generally natural, except that, in a few instances, it is harder than usual in the convolutions. The grey substance contiguous to the pia mater is softened, and reduced to a pulp, to the depth of a quarter or half a line; it is separated into laminæ, of which the external adheres to the pia mater, and the colour of the cortical substance undergoes a peculiar change. All these phenomena are referred by M. Calmeil to inflammation of the cortical substance, which he considers as the proximate cause of the general paralysis of the insane.

*Foville.* This gentleman has still more recently investigated the brain in cases of insanity. In many leading particulars, his observations agree with those of M. Calmeil; but certain appearances, which the latter regards as confined to general paralysis, are connected by Foville with mental derangement. M. Foville has remarked, in the cortical and medullary substances of the brain, various changes of consistence, colour, and nutrition. In the most acute cases of mania, the cortical substance has presented an intensely red colour, approaching to that of erysipelas. Among the chronic changes in this substance, the most frequent is an increased firmness and density of its surface, extending to no great depth, but constituting a distinct lamina, smooth externally, and internally irregular, which, when torn off, leaves the subjacent surface red, soft, and mammillated. The colour of this superficial lamina is considerably paler than natural. M. Foville has noticed morbid appearances, somewhat similar to those just described, in the brains of wild animals which have died in a state of confinement. Among the appearances observed in the medullary substance, one of the most remarkable is an almost fibro-cartilaginous hardness, usually accompanied with a splendid white colour: the colour is not uniformly white, being sometimes yellowish or grey. This induration is conjectured by M. Foville to arise from a matting together of the cerebral fibres by adhesive inflammation. According to this writer, the fibrous mass of the hemispheres is formed by the superposition of several distinct layers of substance, connected by a very fine cellular tissue: these planes, which are easily separable in the healthy state, become inseparable in the induration of the brain accompanying madness. M. Foville found these morbid adhesions in all the cases of general paralysis which he examined, except two.

We must not omit to mention that this anatomist thinks he has traced some morbid alterations of the *nerves*, in connexion with hallucination of the senses: in a female lunatic who had been tormented by optical illusions, the optic nerves were found hard and semitransparent through a great part of their thickness.

The two principal inferences drawn by M. Foville from his researches, are: 1. That morbid changes in the cortical substance are directly connected with mental derangement. 2. That morbid changes in the white substance are directly connected with disorder of voluntary motion. The uniform detection of disease in the cortical substance in cases of general paralysis, induced M. Calmeil to ascribe the loss of muscular power to such disease; M. Foville, however, contends that this conclusion cannot be legitimately derived from the facts, since, in all his cases, although the cortical substance was diseased, there was also induration, serous infiltration, or softening in the white substance, and, in most instances, adhesion of the principal medullary planes to each other.

The sixth chapter is on the theory or pathology of mental derangement.

Notwithstanding the labour and ingenuity that have been devoted to the subject, nothing is yet known of the real proximate cause of insanity; there is, however, one point connected with its pathology which admits of discussion: namely, whether insanity be a disease of the *mind* or of the body. It appears to us, however, not a little extraordinary that there should be two opinions on this subject among men accustomed to think, since the notion of disease of the *mind* is utterly incompatible with either of the possible views of our intellectual constitution. If, according to the doctrine of the materialist, thought is a mere function of the brain, as the biliary secretion is a function of the liver, insanity is merely a disordered state of this function—the soul, as an abstract thinking principle, being out of the question; if, again, there be in man an independent and immaterial thinking principle, of which we, for our own part, have no doubt, it is absurd to talk of this principle being diseased, except as a metaphor, since disease means deranged action in a material organism —at least, this is the only signification we can attach to the term. The notion that the soul or immaterial thinking principle is actually diseased in insanity, though generally abandoned in this country as entirely unphilosophical, has several eminent advocates in Germany, who build their opinion on the unsatisfactory results of anatomical investigations, and the acknowledged influence of moral causes in the production of mental alienation. Professor Heinroth, who adopts this doctrine in its fullest extent, has deduced from it a conclusion as monstrous in morals as it is false in fact—namely, that *moral depravity* is the essential cause of madness! Certainly a most charitable and edifying persuasion, which would make Botany Bay your only lunatic asylum, and a halter the best remedy in inveterate cases! It is much to be wondered at that any man of sense and character should have ventured to promulgate so detestable an opinion. Heinroth has been fully answered by Jacobi, who refers to many examples of insanity occurring in the most pious and excellent individuals, and cites in particular the case of Madame Lavater, the wife of the celebrated physiognomist, a lady remarkable for her amiable character, who, at an advanced period of life, became insane, and continued for a long time in a state of the deepest melancholy. She recovered her reason, however, some months before her death, and evinced all the excellent dispositions which were natural to her.

Dr. Prichard observes that it would be easy to multiply such instances, if it were worth while to contend gravely against the doctrine of Professor Heinroth.

The seventh chapter is on the treatment of insanity, which is, of course, resolved into *therapeutical* and *moral*. On the medical treatment our author is not very diffuse—wherein, we think, he shows his judgment; the subject is one on which very little can be added to what is already known. The principle which should guide our practice is simple. Of the actual state which constitutes insanity we are entirely ignorant; we cannot, therefore, at present hope to strike at the root of the disease; we know, however, that certain morbid conditions of the brain and other organs accompany its progress, and exercise an important influence on the event of the case; to these, therefore, the resources of medicine should be directed, according to the general principles of the science—always keeping in view, however, that we are not dealing with ordinary idiopathic affections, but with the accompaniments of a peculiar and generally a protracted malady. It is by moral treatment that we have most direct control over the mental disorder; but this is a subject which would fill a volume; and being sorely incommoded by lack of space, we must decline entering into it. For the same reason we must be silent on the subjects of puerperal insanity—idiotism—cretinism, and several other points which the reader will find amply and judiciously illustrated in the succeeding chapters.

The tenth chapter contains an accurate survey of the statistics of insanity throughout Europe; and the eleventh of unsoundness of mind in relation to jurisprudence. Under this head are some important observations relating to that form of insanity which our author has denominated *moral,* and whose existence and relation to legal decisions has been too little attended to.

The twelfth and last chapter treats of ecstatic affections, and especially of *animal magnetism.* The curious reader will here find much to his purpose in a full and candid survey of the history of this *pseudo-science.* When we call animal magnetism a pseudo-science, we by no means intend to assert that all the singular observations recorded by its proselytes are without foundation in nature; on the contrary, we believe many of them to be true; and we further think it possible that the investigation of this subject may lead to some interesting discoveries in the physiology of the nervous system; but when we hear of all the incredible phenomena of *clairvoyance* in its highest degree, in which the illuminated individual is enabled to outstep the limits of time and space, to penetrate the veil of nature, to dive into futurity, and to ascertain all that other people are doing, or ever will do, we must declare, in the words of Samuel Johnson, that "attention recoils from the repetition of a tale, which, when it was first heard, was heard with scorn." Philosophical toleration should be ample, but still it must have limits; and when doctrines are advanced repugnant to the plainest dictates of common sense, there is no illiberality in passing them by, as unworthy of attention. If animal magnetism be true to the extent that many believe, it must become an object of legislation as well as science; and we see no remedy for the evils of exalted magnetism but the horribly Draconic one of putting to death all *clairvoyans,* as persons entirely unfit for a mundane state of things, and whose existence is incompatible with the well-being of society. An inquisitive man, who pries into everything, and tells tales, is a most pernicious individual; but one who detects the present and future proceedings of all the world, is many degrees more dangerous than a mad dog; "hunc tu Romane caveto."

Several interesting subjects in Dr. Prichard's treatise we have been obliged to pass by without even alluding to them; but we regret our omissions the less, because we can conscientiously recommend the book to the reader's most attentive perusal. Though inferior to the works of Haslam in depth of thought and felicity of style, it is nevertheless the most elaborate, comprehensive, and useful treatise on insanity that has yet appeared in this country.

